# Capacity Building in Operational Research: More than One Way to Slice the Cake

**DOI:** 10.3389/fpubh.2015.00176

**Published:** 2015-07-09

**Authors:** I. D. Rusen, Anthony D. Harries, Rony Zachariah, Andy Ramsay

**Affiliations:** ^1^International Union Against Tuberculosis and Lung Disease, Paris, France; ^2^Médecins Sans Frontières, Brussels, Belgium; ^3^World Health Organization, Geneva, Switzerland

**Keywords:** training, operational research, methodology, mentorship, programmatic

The importance of programmatically relevant operational research (OR) as a key driver to strengthen public health activities is well recognized (1). Moreover, building capacity of health workers from high-disease burden countries to independently conduct OR on priority issues within their health services is accepted as an essential component of public health programs. However, the approach taken in building local OR capacity varies widely between training initiatives. Program managers and those responsible for planning OR training activities may thus be left asking themselves what the best approach is for their own setting?

The Union-MSF model is the training model adopted by the Structured Operational Research and Training Initiative (SORT IT), a global partnership led by the Special Program for Research and Training in Tropical Diseases at the World Health Organization (2). It has set the standard for hands-on, milestone-based training with an emphasis on tangible outputs – marked largely by the submission of research papers to peer-reviewed scientific journals. The success of this approach is undeniable as evidenced by the research outputs: by March 31, 2015, there had been 247 papers submitted to peer-review journals from 20 completed OR courses of which 208 (84%) were in press or published. The involvement in SORT IT has further established the standardization and quality of this training. To date, most of the research conducted under SORT IT has been retrospective analyses of routinely collected data. SORT IT partners are now planning expansion into more complex, prospective operational, and implementation research using mixed quantitative and qualitative methods.

The International Union Against Tuberculosis and Lung Disease (The Union)-implemented TREAT TB project has undertaken several OR training activities within the multi-year research initiative (3). Several India-based trainings within this Initiative have followed very closely the SORT IT approach. TREAT TB has also supported training in South Africa, again adopting modular, hands-on approaches to research protocol development and implementation. However, the South Africa training program (Operational Research Assistance Project) included participants from both health services and academic settings, who were linked in pairs to jointly undertake a research project. Once again, the development and submission of a research paper related to a priority issue within their health service was a key output of the effort. More recently, TREAT TB has developed a virtual training program for OR. Utilizing a combination of synchronous (online learning together simultaneously) and asynchronous approaches, the program attempted to increase access to OR training while allowing participants to remain in their communities and workplaces. A small group of participants was selected for the pilot course. Four participants (out of six initiating the training) completed the training and presented their findings at an international scientific conference in 2014. Publication of their research manuscripts is pending.

A country-based OR training program in Ethiopia was jointly conducted by international and local partners over a 2-year period beginning in 2012. Again utilizing modular training techniques, the Ethiopian Operational Research Initiative implemented a team approach to the research development and implementation phase.

Teams usually consisted of four people representing the regional health bureau (RHB), the regional laboratory, a health facility and a local university. The research topics selected were based on a national OR priority setting exercise to ensure the relevance of the research undertaken. Successful outputs of the research effort included presentations in a symposium at an international conference and publication of a special supplement of a peer-reviewed journal comprising the research outputs from the initial wave of participants (4).

These highlighted training initiatives were not without challenges. Hurdles in securing ethics approval locally and unexpected barriers to access data during the research implementation phase were among the “real-life” research issues faced by participants in the programs. The availability of experienced mentors within the course faculty offered timely support and, in most instances, practical solutions to overcome these challenges.

The overall success of these varied OR training efforts suggests a range of approaches might attain the desired goal of building a larger pool of health workers able to independently undertake locally relevant OR leading to improved health services. While the experiences highlighted were focused on participants from low-to middle-income settings, the approach taken is likely equally applicable to public health workers of all backgrounds. Ongoing evaluation of OR training initiatives is essential to better delineate the ideal package for a given setting. Important components to be evaluated to direct future training initiatives include: the role of online/virtual training methods, the minimum duration of training required to transfer the necessary research knowledge and skills, the scope of research methods, and approaches appropriate for training different levels of health workers. Nonetheless, in advance of further evaluations, common factors that appear to form the basis of a successful research training program include: clear, time-based milestones for each stage of the research process, a strong mentorship component and concrete research outputs – namely a successfully completed research manuscript that is considered of sufficient quality to warrant submission to a national or international peer-reviewed journal. In addition to these core components for all OR trainings, we recommend an expansion of OR training content to include advanced research methods in the form of specialized training on qualitative research methods, health economics and how to conduct systematic reviews with meta-analyses among others, along with further evaluation of these efforts. Additionally, moving forward, an emphasis on assessment of the impact of OR on program practice and policy will be an increasingly important consideration in gaging the success of research training efforts. Ministries of Health and other stakeholders keen on increasing local capacity for OR, yet concerned about selecting a single, “correct” approach, may be reassured that a variety of approaches tailored to their local setting may deliver the ultimate goal of strengthened public health programs, provided the essential components are not overlooked.

## Conflict of Interest Statement

The authors declare that the research was conducted in the absence of any commercial or financial relationships that could be construed as a potential conflict of interest.

## References

[1] Harries AD Rusen ID Reid T Detjen AK Berger SD Bissell K The union and Médecins Sans Frontières approach to operational research. Int J Tuberc Lung Dis (2011) 15:144–54. 21219672

[2] Ramsay A Harries AD Zachariah R Bissell K Hinderaker SG Edginton M The structured operational research and training initiative for public health programmes. Public Health Action (2014) 4(2):79–84. 10.5588/pha.14.0011 PMC4539036 26399203

[3] The Union. TREAT TB: Description of Research Outputs (2014). Available from: http://www.theunion.org/what-we-do/publications/technical/treat-tb-description-of-research-outputs

[4] Klinkenberg E Assefa D Rusen ID Dlodlo R Shimeles E Kebede B The Ethiopian initiative to build sustainable capacity for operational research: overview and lessons learned. Public Health Action (2014) 4(Suppl 3):S2–7. 10.5588/pha.14.0051 PMC4542073 26478509

